# Interpretable Clinical Decision-Making Application for Etiological Diagnosis of Ventricular Tachycardia Based on Machine Learning

**DOI:** 10.3390/diagnostics14202291

**Published:** 2024-10-16

**Authors:** Min Wang, Zhao Hu, Ziyang Wang, Haoran Chen, Xiaowei Xu, Si Zheng, Yan Yao, Jiao Li

**Affiliations:** 1 Institute of Medical Information/Library, Chinese Academy of Medical Sciences & Peking Union Medical College, Beijing 100020, China; wang.min@imicams.ac.cn (M.W.); chen.haoran@imicams.ac.cn (H.C.);; 2 Chinese Academy of Medical Sciences & Peking Union Medical College/National Center for Cardiovascular Diseases, Fuwai Hospital, Beijing 100037, China; hu_zhao95@126.com

**Keywords:** ventricular tachycardia, machine learning, etiological diagnosis, Explainable Artificial Intelligence, clinical decision-making

## Abstract

**Background:** Ventricular tachycardia (VT) can broadly be categorised into ischemic heart disease, non-ischemic structural heart disease, and idiopathic VT. There are few studies related to the application of machine learning for the etiological diagnosis of VT, and the interpretable methods are still in the exploratory stage for clinical decision-making applications. **Objectives:** The aim is to propose a machine learning model for the etiological diagnosis of VT. Interpretable results based on models are compared with expert knowledge, and interpretable evaluation protocols for clinical decision-making applications are developed. **Methods:** A total of 1305 VT patient data from 1 January 2013 to 1 September 2023 at the Arrhythmia Centre of Fuwai Hospital were included in the study. Clinical data collected during hospitalisation included demographics, medical history, vital signs, echocardiographic results, and laboratory test outcomes. **Results:** The XGBoost model demonstrated the best performance in VT etiological diagnosis (precision, recall, and F1 were 88.4%, 88.5%, and 88.4%, respectively). A total of four interpretable machine learning methods applicable to clinical decision-making were evaluated in terms of visualisation, clinical usability, clinical applicability, and efficiency with expert knowledge interpretation. **Conclusions:** The XGBoost model demonstrated superior performance in the etiological diagnosis of VT, and SHAP and decision tree interpretable methods are more favoured by clinicians for decision-making.

## 1. Introduction

Ventricular tachycardia (VT), a common arrhythmic disorder, often serves as an inaugural or solitary manifestation across the spectrum of structural heart diseases [1], typically accompanied by severe hemodynamic disturbances during episodes. Of significant concern, sustained VT represents a pivotal contributing factor to sudden cardiac death, with a reported annual toll of 544,000 fatalities in mainland China alone [2], corresponding to an estimated incidence of around 50 cases per 100,000 person–years within middle-aged populations [3]. Etiological diagnosis is one of the key aspects in the diagnosis and treatment of VT. The multifaceted origins of VT can be systematically classified into ischemic heart diseases, non-ischemic structural heart diseases, and idiopathic VT [3,4,5,6]. Notably, the first two categories fall under the umbrella of structural heart pathologies. With advancing age, there is a heightened prevalence of structural heart disease, particularly ischemic heart disease, thereby correspondingly augmenting the incidence of VT related to structural abnormalities. In contrast, idiopathic VT characterises those cases where VT arises in the absence of structural heart disease, comprising roughly 10% of all VT occurrences and being more frequent among younger and middle-aged individuals [7]. There are two differential diagnosis strategies commonly used in clinical practice which can guide the subsequent management, i.e., ischemia and non-ischemia and idiopathic versus non-idiopathic (structural). These dichotomies are predicated on the understanding that when VT stems from ischemic heart disease, treatment focuses on addressing the underlying coronary artery pathology; conversely, in cases of non-idiopathic VT, intensified efforts are directed at identifying potentially treatable substrates. Consequently, a tripartite categorisation—idiopathic VT, ischemic heart disease, and non-ischemic structural heart disease—is anticipated following an initial assessment. However, despite these strategies, the rate of accurate etiological diagnosis for VT remains suboptimal [8]. Clinical decision-making regarding VT can pose considerable challenges due to the wide array of potential underlying conditions, varied clinical presentations, and the inherent risk of hemodynamic instability requiring immediate life-sustaining interventions [5,9]. This complexity underscores the need for improved diagnostic tools and strategies to enhance the precision and timeliness of etiological diagnoses in VT patients.

Clinical Decision Support Systems (CDSS) are indispensable tools in the clinical decision-making process, playing a crucial role in optimising diagnostic and treatment procedures and enhancing the precision of decisions. Currently, mainstream CDSS are primarily divided into two major categories based on their operational mechanisms: knowledge-driven and data-driven [10]. Knowledge-driven systems rely on authoritative Clinical Practice Guidelines (CPG) and a wealth of medical literature, with an integrated, comprehensive medical knowledge base within the system. However, knowledge-driven CDSS face certain challenges in model innovation, and the potential of the algorithms is constrained by the specialised domain knowledge, making it difficult to fully express some complex medical knowledge with generic computer programs, which has led to a relative scarcity of research in this area in recent years. Meanwhile, an increasing number of researchers are shifting their focus towards exploring data-driven clinical decision-making models in the hope of discovering new breakthroughs.

Machine learning (ML), as an artificial intelligence technology, has seen remarkable proliferation and efficacy in the medical domain, with extensive research substantiating its value in bolstering clinical decision-making for cardiovascular diseases [11,12]. It has led to breakthroughs in areas such as diagnosis, risk stratification, and rapid detection of abnormal heart rhythms within intensive care units through seamless integration with electronic health records [13,14]. However, despite these developments, fewer artificial intelligence-based decision support tools have been implemented in the field of cardiovascular disease due to numerous challenges encountered during the transition from algorithm development to practical application [15]. Specifically, following the confirmation of VT through electrocardiogram (ECG), clinicians still spend considerable time determining the underlying causes of VT in patients to devise subsequent treatment and medication plans. The incorporation of efficacious AI technologies into the etiological diagnosis of VT would significantly streamline clinical decision-making, thereby enhancing the efficiency of healthcare professionals.

For clinicians, the insights and justifications provided by AI models are more critical than the technical details of their implementation [16]. The limited adoption of AI-based clinical decision-making tools is largely attributed to the need for interpretability; healthcare providers tend to favour knowledge-driven systems that rely on well-established clinical guidelines and expert consensus. Such knowledge-driven systems use pre-programmed rule engines and algorithms to provide instant, targeted diagnostic hints, treatment suggestions, and risk alerts based on inputted patient data, ensuring medical decisions adhere to the highest standards of evidential support and professional agreement. Their inherent interpretability makes them more likely to earn clinicians’ trust. Explainable Artificial Intelligence (XAI) emerges as a crucial subfield of ML, aiming to render AI models and their decision-making transparent and understandable [17]. However, while XAI techniques have matured significantly in computer science, their application to clinical decision-making may still present interpretability gaps [18]. Despite burgeoning interest in XAI research over the past few years, human-centred XAI approaches remain in their infancy. Clinicians might harbour scepticism towards the outputs of these tools or find them difficult to integrate into daily practice [19]. For clinicians, understanding AI methodologies presents several challenges, including (1) lack of technical knowledge: unlike technical users, clinicians typically do not possess specialised expertise in artificial intelligence, machine learning, data science, or programming; (2) adaptation to clinical variability: the development of XAI for clinicians must accommodate the variability of clinicians, clinical settings, and decision support needs. For instance, when using AI as a diagnostic aid, doctors might require different types of explanations from the AI system; and (3) human-centred approach: as ultimate decision-makers, clinicians need an XAI method that provides them with various interpretable approaches to choose from according to their preferences.

In recent years, researchers have started to explore clinician-centred XAI methods designed explicitly to facilitate and enhance clinical decision-making processes [20,21]. Efforts are underway to build AI models that combine high accuracy with strong interpretability akin to knowledge-driven models. In addition, a comprehensive and standardised set of assessment protocols for evaluating the interpretability of machine learning methods in this context is currently lacking. To bridge these gaps, this paper constructs an interpretable machine learning model for VT etiological diagnosis and compares it with the traditional knowledge-driven model based on a designed clinical decision-oriented interpretable evaluation protocol to assess the interpretability of the machine learning methods.

## 2. Materials and Methods

**Figure 1:**
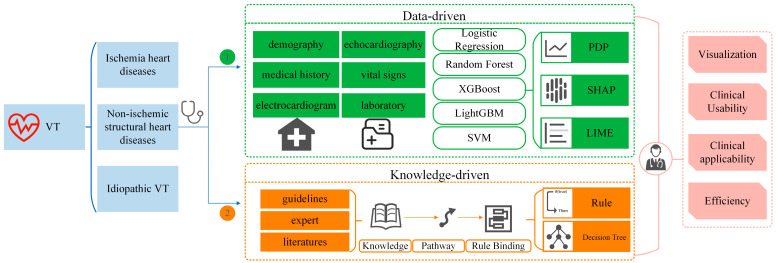
A flowchart of the interpretable machine learning model and clinical application.

Figure 1 shows a flowchart of the construction of an interpretable machine learning model oriented towards the etiological diagnosis of VT and its clinical application.

### 2.1. Data Collection and Preprocessing

**Figure 2:**
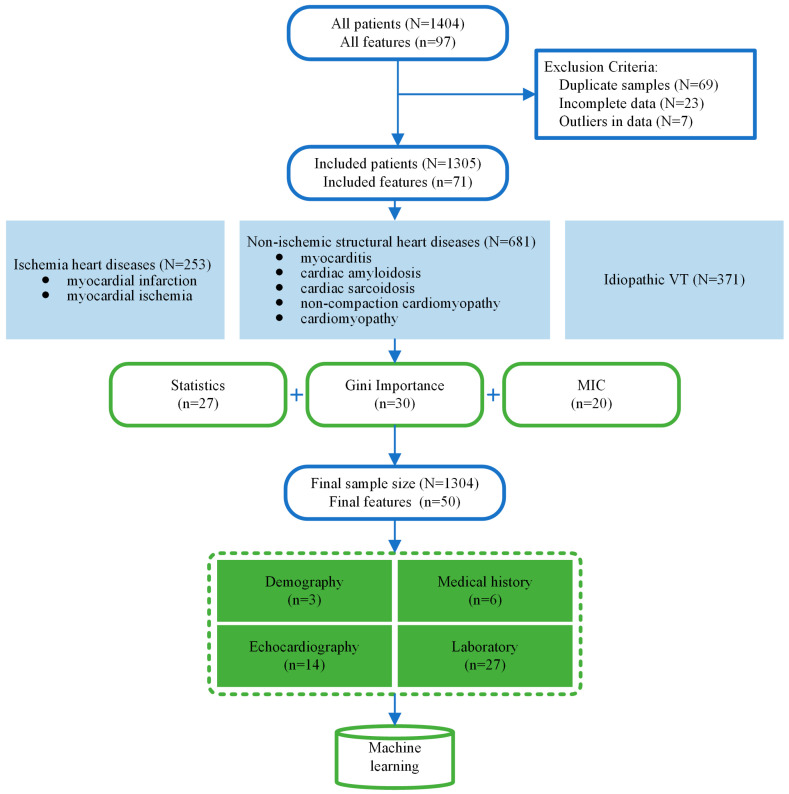
Data processing and feature selection.

The retrospective data that was used for training the machine learning algorithms was provided by the Fuwai Hospital, CAMS, China. Patients with ventricular tachycardia at the Arrhythmia Centre of Fuwai Hospital were consecutively admitted between 1 January 2013 and 1 September 2023. The inclusion criterion was as follows: patients with a discharge diagnosis that included “ventricular tachycardia”. The patients’ records comprised their clinical data, including medical history, vital signs, current medications, electrocardiograms (ECGs), echocardiograms, and laboratory test results, all of which were diagnosed by professional physicians. The VT due to ischemia heart diseases were defined as whose diagnosis contained “myocardial infarction” or “myocardial ischemia”. The VT due to non-ischemic structural heart diseases contained “myocarditis”, “cardiac amyloidosis”, “cardiac sarcoidosis”, “non-compaction cardiomyopathy”, or “cardiomyopathy”. The idiopathic VT is defined according to the discharge diagnosis as “idiopathic ventricular tachycardia” (Figure 2). All the etiological diagnosis label was reviewed by the cardiologists, which is regarded as the golden standard.

Datasets based on real-world clinical practice, such as our dataset of 1305 patients, have a number of missing values and outliers, if the missing value proportion exceeds 30%, the variable shall be deleted; otherwise, K-Nearest Neighbours Imputation (KNN) is employed to fill in the missing data. The KNN imputation method estimates missing values by calculating the weighted average of attribute values from the K nearest known data points, where the proximity of these points is determined based on their distances from the point with the missing value.

In the feature selection phase, we screened features using three methods (Figure 2). Firstly, by using a statistical test method to find out those features which are significantly associated with the target variable using the Gini importance within the random forest model and selecting the features with top-ranked Gini coefficients. Meanwhile, MIC is employed to quantify both correlation and redundancy among features, with results closer to 0, signifying weaker associations between them, according to the results of MIC. The features whose MIC value with the target variable exceeds the set threshold are selected. Finally, the set of features screened by these three methods is taken as a concatenated set, which combines the advantages of multiple feature selection methods to cover all the features that have an important impact on the model as comprehensively as possible.

### 2.2. Machine Learning Model Construction

In order to develop a model for the etiological diagnosis of VT, we applied 5 different ML models for supervised learning (implementation in the Python Scikit-Learn(version 1.1.3), https://scikit-learn.org/stable/): (1) Logistic regression. (2) Random forest [22]. (3) XGBoost (version 1.7.6) [23], a boosting method which provides gradient boosting on an ensemble of many decision trees. (4) Light Gradient Boosting Machine (LightGBM, version 4.0.0) [24], which introduces two new techniques based on the traditional Gradient Boosting Decision Tree (GBDT): gradient one-sided sampling and independent feature merging. (5) Support Vector Machine (SVM) [25], which creates a decision boundary between two classes that enables the prediction of labels from one or more feature vectors. The model was constructed using GridSearchCV (version 1.1.3) to optimise the hyperparameters. We used GridSearchCV to optimise the hyperparameters. We specifically tuned the following key hyperparameters: n_estimators, the number of decision trees; max_depth, the maximum depth of each tree; min_samples_split, the minimum number of samples required for internal node repartitioning; min_samples_leaf, the minimum number of samples required for leaf nodes; and max_features, the number of features considered in finding the optimal split.

Considering the metrics for VT etiological diagnosis, we used a weighted macro-average approach to calculate assessment metrics to solve the triple classification problem. We used the precision, recall, and F1-score derived from the ten-fold cross-validation as model evaluation criteria, as shown in Equations (1)–(3). Precision refers to the proportion of true positive samples among all samples predicted as positive by the model. It reflects the accuracy of the model’s positive predictions. Recall (also known as sensitivity) is the proportion of true positive samples that are correctly identified as positive among all actual positive samples. It reflects the model’s ability to identify positive cases. The F1-score is the harmonic mean of precision and recall, providing a balanced measure for evaluating the overall performance of the model. The macro-average is the arithmetic mean of the performance metrics of the classification model on each category, without considering the number of samples in each category. However, in practical applications, the number of samples in different categories can vary significantly. Directly using macro-average may lead to an overemphasis or neglect of categories with smaller sample sizes. Therefore, we adopt the weighted macro-average method, which takes into account the proportion of samples in each category, providing a more balanced and representative overall performance measure. The weighted macro-average is calculated by multiplying the precision, recall, and F1-score of each category by its respective sample size and then dividing by the total number of samples. This ensures that the performance of each category is weighted according to its importance in the dataset, resulting in a more accurate representation of the model’s overall performance. The sequence of Equations (4)–(6) is presented below, where C is the total number of categories and $N_{i}$ is the number of samples in the ith category.

(1) $${Precision}_{c}=\frac{{TP}_{c}}{{TP}_{c}+FP_{c}}$$

where ${TP}_{c}$ denotes the number of true instances of category c, and $FP_{c}$ denotes the number of false-positive.

(2) $${Recall}_{c}=\frac{{TP}_{c}}{{TP}_{c}+{FN}_{c}}$$

where ${FN}_{c}$ denotes the number of false counterexamples for category c.

(3) $$F_{1}-score=2\times \frac{{Precision}_{c}\times {Recall}_{c}}{{Precision}_{c}+{Recall}_{c}}$$

(4) $${Precision}_{w\_macro}=\frac{\sum_{i=1}^{C}N_{i}P_{i}}{\sum_{i=1}^{C}N_{i}}$$

(5) $${Recall}_{w\_macro}=\frac{\sum_{i=1}^{C}N_{i}R_{i}}{\sum_{i=1}^{C}N_{i}}$$

(6) $${F_{1}-score}_{w\_macro}=\frac{\sum_{i=1}^{C}N_{i}{F1}_{i}}{\sum_{i=1}^{C}N_{i}}$$

### 2.3. Interpretable Methods and Evaluation

#### 2.3.1. Knowledge-Based

In knowledge-driven systems, rule-based models are widely recognised for their dedication to simulating the decision-making processes of human experts and achieving significant results. To thoroughly evaluate the consistency of interpretable methods in machine learning with expert knowledge, that is, interpretability, this study utilised the clinical pathways established in a previous project for a partial arrhythmia diagnosis and treatment model. We have developed a simplified prototype of diagnostic rules for the causes of ventricular tachycardia, which are derived from evidence-based knowledge sources such as clinical guidelines, expert consensus, and medical literature. Specifically, this includes designing a set of clinical pathways suitable for the clinical diagnostic and treatment process of arrhythmias, clarifying key decision points and operational standards at each stage of the pathway, and constructing corresponding rules based on evidence-based medicine. Ultimately, these rules were bound to the corresponding nodes of the clinical pathways. This rule prototype is closely related to the human reasoning process and can be visually presented in two forms: rule text and decision trees. Rules are based on causal logic chains of the form “IF...THEN...”, which associate a predefined set of conditions with a corresponding decision outcome. Decision trees represent decision conditions through branching nodes, and leaf nodes represent the final decision outcome.

#### 2.3.2. Machine Learning Interpretable Methods

The Partial Dependence Plot (PDP) [26] shows the marginal effect of one or two features on the predictions of a machine learning model. Partial Dependence Plots can show whether the relationship between targets and features is linear, monotonic, or more complex. A partial dependency graph is a global approach, as it considers all instances and gives a global description of the relationship between the features and the predicted results. Individual Conditional Expectation (ICE) [27] shows a line for each instance that shows how the prediction of the instance changes when the feature changes, equivalent to the PDP for a single data instance.

Local interpretable model-agnostic explanations (LIMEs) [28] focus on training local agent models to explain individual predictions. It can be expressed as in Equation (7):

(7) $$explanation\left(x\right)=arg\underset{g\in G}{\min}\;L\left(f,g,\pi_{x}\right)+\Omega (g)$$

As an example of an interpretable model, x is model g (e.g., a linear regression model), with minimisation loss L (e.g., mean square error) measuring how close the interpretable g is to the prediction of the original model f, while model complexity (g) is kept low (e.g., fewer features are preferred). G is the family of possible explanations, e.g., all possible linear regression models. The proximity $\pi_{x}$ defines the size of the neighbourhood around the instance x when we consider the explanation.

SHAP (SHapley Additive ExPlanations), based on the concept of Shapley values in cooperative game theory [27], is an additive method used to calculate the contribution of each feature to the model predictions, which can be used to interpret the feature importance of the model predictions. SHAP specifies the interpretation as in Equation (8):

(8) $$g(z^{\prime })=\phi_{0}+\sum_{j=1}^{M}\phi_{j}z_{j}^{\prime }$$

where g is the interpretable model, $z^{\prime }\in {\left\{0,1\right\}}^{M}$ is the coalitional quantity, M is the maximum coalition size, and $\phi_{j}\in R$ is the feature attribution Shapley value of the feature.

Studies have been conducted on the visualisation forms of interpretable methods, identifying 3 categories of visualisation methods, with a total of 12 user-friendly forms [29]. To compare the results with traditional knowledge-driven interpretable results (decision rules and decision tree), we selected 4 main interpretable methods: PDP, ICE, LIME, and SHAP.

Evaluating Explainable Artificial Intelligence (XAI) presents challenges [30], as there is currently no unified standard. This study will establish an assessment framework for interpretable machine learning models tailored to clinical decision-making by referring to existing XAI evaluation guidelines [31], encompassing the following dimensions: visualisation, clinical usability, clinical applicability, and efficiency. For a more comprehensive and professional evaluation, we adopted a semi-structured interview method, which included 10 cardiologists who use the hospital information management system on a daily basis and have rich experience in diagnosing VT and another 5 experts in the field of medical informatics, who have an in-depth understanding of the needs and pain points of the existing medical decision support tools. We designed a questionnaire that included both single-choice and textual discourse questions that covered the predefined evaluation metrics (visualisation, clinical usability, and clinical applicability). Specifically, we asked the experts to rate each evaluation metric and provide detailed feedback. Each expert rated each evaluation metric on a scale from 1 to 5. Additionally, the experts provided textual feedback to elaborate on their rating reasons. We aggregated all the experts’ ratings and calculated the average score for each evaluation metric. For the open-ended questions, we conducted a qualitative analysis to extract key opinions and suggestions. Finally, based on the average scores and the qualitative feedback, we assigned weights to each evaluation metric.

Visualisation. The visualisation form of interpretable methods should be easy for clinicians to comprehend without requiring technical expertise. Visualisation forms are divided into text, pictures, diagrams, and other forms. In the clinical decision-making of VT etiological diagnosis, the visualisation form of the interpretable method is very critical, which helps clinicians to better understand and trust the decision-making process of the model. A good visual form should have (1) the volume of information should be moderate to avoid too much information leading to visual confusion, and at the same time, it should fully demonstrate the key features and interrelationships; and (2) the difficulty level of the visual information should be appropriate to the clinician’s level of knowledge, which makes it easy for the clinician to quickly grasp the core points.

Clinical usability. The usability of the interpretable method in a clinical decision-making scenario should align with and contribute value to clinicians’ clinical decision patterns. We compare interpretable machine learning methods with expert knowledge-based models (decision rules and decision tree) to assess whether the interpretation of decision-making by interpretable machine learning methods is consistent with expert knowledge.

Clinical applicability. Primarily, this assesses the applicability of the interpretable method and its visualisation approach within specific clinical scenarios. Interpretable methods are categorised into global and local methods, and depending on different requirements, these scenarios may include population-level (global) and person-level (local) [32].

Efficiency. Here, efficiency does not refer to the computational efficiency of the model itself but rather the time and efficiency spent by clinicians when using the potentially best-suited interpretable method selected based on the previous three evaluation criteria. Specifically, we will measure the time required for a clinician to make a final diagnostic decision using an interpretable method in actual clinical practice. We will calculate the time it takes for a clinician to utilise one such explainable method to facilitate a single instance of a real-world clinical decision-making process.

#### 2.3.3. Statistical Methods

Statistical analyses were performed using Python (version 3.9), and the differences were considered statistically significant at *p* < 0.05. For continuous variables such as age, BMI, and other measures that conformed to a normal distribution, we expressed the data as means. The t-test was used for comparison between groups, which is a widely accepted method for comparing the means of two independent groups when the data are normally distributed. For categorical variables such as sex and comorbidities, the data were expressed as frequencies (%). The χ^2^ (chi-squared) test was used for comparison between groups. The χ^2^ test is appropriate for categorical data and is used to determine whether there is a significant association between two categorical variables.

## 3. Result

### 3.1. Patient Population

Of all the 1305 patients with VT, non-ischemia structural heart diseases ranked first, at nearly half of the amount (681 patients), suggesting that non-ischemic structural heart disease is a major cause of VT. Idiopathic VT was in the middle, at about 30% (371 patients). Ischemia heart diseases were the least, at only 20% (254 patients). The mean age of enrolled patients was 42 years old, which suggests that VT is not limited to the elderly population, but younger patients are also affected. The males consisted of the majority, at 80%. The most prevalent comorbidity was hypertension, which was as high as 23.26%. The mean LVEF was 56.84%, but 12.4% of the patients had an LVEF below 40%, suggesting possible serious cardiac dysfunction (Table 1).

### 3.2. Performance of Etiological Diagnosis Prediction

The overall performance of the VT etiological diagnostic model is shown in Table 2, and the XGBoost model achieved better performance in terms of the evaluation metrics precision, recall, and *F*1 on the three ventricular tachycardia etiological diagnostic works of interest, with *F*1 reaching 89.7%, 76.9%, and 94.1% of the experimental performance.

Table 3 shows the overall model performance of five models. The XGBoost model achieved the best performance on the VT etiological diagnosis (precision, recall and F1 scores of 88.4%, 88.5%, and 88.4%, respectively).

### 3.3. Interpretation and Visualisation

For the interpretable analysis of VT etiological diagnosis, we show two ways of interpretation based on expert knowledge (decision rules and decision tree) and the results of 4 interpretable machine learning methods (PDP, ICE, LIME, and SHAP).

**Figure 3:**
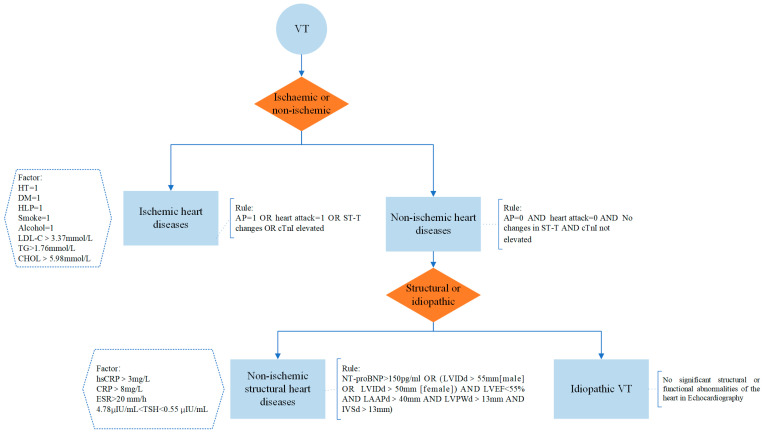
Knowledge-based prototype.

Figure 3 shows prototype decision rules and a decision tree for the expert knowledge-based etiological diagnosis of VT, where the factor boxes indicate the relevant factors that may affect the outcome of that decision. Here, we aim to compare it only with an interpretable machine learning approach in terms of visualisation and clinical usability dimensions, so a prototype of the decision interpretation is easily drawn based on the knowledge from cardiologists.

**Figure 4:**
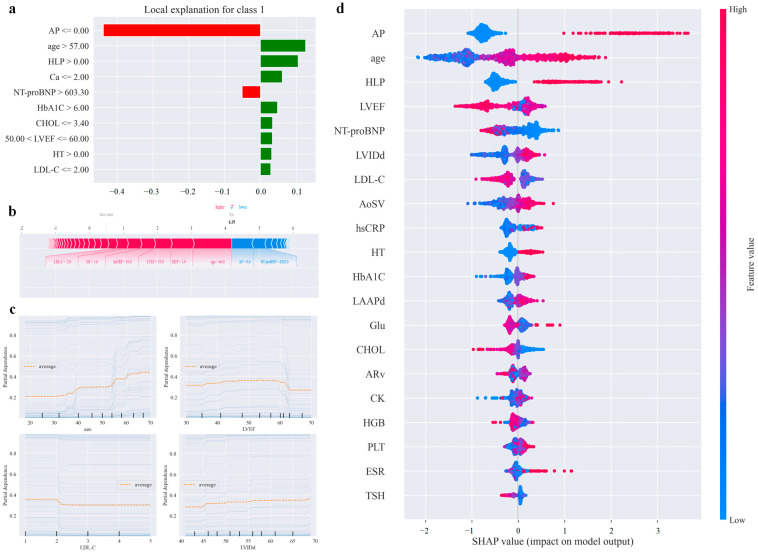
Interpretable analysis results. Notes: (**a**): LIME plots, where the red bars indicate features that have a negative effect on category predictions, and the green bar indicates that the feature is a positive influence on the category prediction; (**b**): SHAP explanation force plot; (**c**): PDP and ICE plots; and (**d**): SHAP summary plot.

PDP and ICE show the relationship between each feature and the target outcome. We selected plots of the outcomes of the four features with a diagnosis of ischaemic heart disease, and the yellow line shows the PDP of age, LVEF, LVIDd, and LDL-C on the etiological diagnosis of VT. The blue line shows the ICE of the probability of risk of death for individuals with this characteristic (n = 50). As shown by the graph, the risk of a patient being judged to have ischemic heart disease increases progressively with age between 55 and 60 years old (Figure 4c). The probability of diagnosing ischaemic heart disease became lower when the LVEF was around 60%. The interpretation of the features and diagnostic results by the PDP and ICE plots was broadly in agreement with expert knowledge in terms of trends but differed in terms of thresholds at key nodes (e.g., expert knowledge considered that the probability of diagnosing ischaemic heart disease was lower when the LVEF < 55%).

The SHAP summary plot shows the ranking of the importance of all input features in the etiological diagnosis of VT. Each point on the plot is a Shapley value for a feature and an instance of it, with colours representing feature values from low to high. As shown, angina pectoris and age are the most important features influencing the etiological diagnosis of VT. The higher the age, the greater the risk of ischemic heart disease (Figure 4d). The results of this interpretable method are broadly consistent with expert knowledge for the diagnosis of ischaemic heart disease, and there is no contradiction of judgements.

Since the LIME and SHAP explanation force plots are local interpretable methods, we evaluated both interpreters in one randomly selected instance correctly judged to be ischemic heart disease. LIME shows that the characteristics of this patient correctly predicted as ischemic heart diseases included no angina being present, age > 57 years, and there was a history of hyperlipidaemia and hypertension. The interpretation of these features is broadly consistent with expert knowledge, but the analysis of critical thresholds differs from the results of expert knowledge, and the LIME graphs show some features that expert knowledge would not be concerned with (Figure 4a). The SHAP explanation force plot shows that this patient had a high risk of being predicted to have ischemic heart diseases, and it shows the specific data of the patient, where the red arrow on the left side indicates the contribution to the model output when the feature value is high, the blue arrow on the right side indicates the contribution to the model output when the feature value is low, and the direction of the arrow suggests the influence of the feature on the prediction result. According to the local interpretation of the results, age, HLP, and LVEF were the features that contributed more to the model’s prediction of the etiological diagnostic outcome for this patient, and the thresholds for the relevant features remained largely consistent with expert knowledge (Figure 4b).

### 3.4. Evaluation of the Clinical Application of Interpretable Methods

Table 4 shows the evaluation of several interpretable methods in the dimensions of visualisation, clinical usability, and clinical applicability. Decision rules can assist with initial clinical reasoning, and the textual form of the rule is simple to understand without the need to add explanations to the interpretation. However, rules do not cover complex diseases and lack information about the credibility of the decision to show the full picture of the decision. The tree visualisation form of a decision tree is more in line with the actual diagnosis and treatment judgement process of clinicians, distinguishing different types through different colours and shades, but the judgement of complex clinical decisions may require a deeper tree structure. The granularity of PDP and ICE methods is the deepest, and visual interpretations are developed for each feature in relation to the target outcome. High number of visualisations, which is time-consuming for complex disease judgements. However, due to the large number of visualised images, it is more time-consuming to judge complex diseases. LIME is better at interpreting clinical decisions for individual patients, providing bar graphs about the contribution of individual patient characteristics to the target structure, but has less depth of interpretation for complex decisions. SHAP is applicable to a wide range of clinical scenarios, and the visualisation is rich in form, representing both feature importance and feature effects. However, the visualisations have more information elements, and clinicians may require guidance.

### 3.5. Feature Ablation Study

In order to further verify the specific contribution degree of features, we implemented a Feature Ablation Study. Based on the SHAP value to rank the importance of the features (Figure 4d), we removed the features according to their importance in descending order, retrained the XGBoost model while keeping the rest of the parameters unchanged, and compared the performance of the model after removing the features with that of the original model. Table 5 shows the results of the feature ablation experiments compared to initial performance (precision, recall, and F1 scores of 88.4%, 88.5%, and 88.4%, respectively), and we found that even after removing these important features, the performance of the model only showed a relatively small change without significant degradation, which suggests that the model still maintains a better prediction ability in the absence of a certain feature, and the model is not free from the risk of overfitting.

## 4. Discussion

In the realm of clinical decision-making, the twin concerns of validity and interpretability are widely recognised as paramount challenges that XAI must effectively confront and resolve [33]. In this study, we constructed an XAI framework for the etiological diagnosis of VT, demonstrating commendable performance in both validity and interpretability.

Ventricular Tachycardia (VT) is a severe arrhythmia associated with various factors, including structural heart disease, ischemic or non-ischemic cardiomyopathy, and idiopathic causes. Therefore, it has become particularly important to develop effective clinical decision support models to assist VT diagnosis. In recent years, significant progress has been made in the prevention and treatment of cardiovascular diseases. However, VT remains a major issue for many patients with heart disease. Currently, research on the etiological diagnosis of VT is relatively limited, and most studies still rely on evidence-based medicine and traditional statistical methods [34]. For example, a series of guidelines published by the European Society of Cardiology provides recommendations based on these approaches [3]. Additionally, a retrospective study used a Cox regression model to predict the incidence of VT events in patients with arrhythmogenic right ventricular cardiomyopathy (ARVC, a rare inherited nonischemic cardiomyopathy characterised clinically by the occurrence of fatal arrhythmic events) [35]. Another study employed a Bayesian survival model to analyse the effectiveness of ablation versus antiarrhythmic drug therapy in VT [36]. Although these studies have improved our understanding of VT diagnosis and treatment to some extent, they primarily use traditional methods, which lack the ability to effectively mine complex data patterns. Some initial attempts have been made to apply machine learning to VT diagnosis and prediction. For instance, one study designed an ML model to predict the recurrence of premature ventricular complex (PVC) and idiopathic ventricular tachycardias (IVT) after radiofrequency ablation [37]. However, this study found no significant variables affecting VT recurrence and suggested the need for further optimisation and fine-tuning of the machine learning model to enhance its clinical utility.

In the etiologic diagnostic strategy for VT constructed in this paper, the XGBoost model demonstrated better results (Table 3), which is in line with the daily diagnostic thinking of clinicians. The model we developed takes into account the current clinical state of the patient, and the clinical scenario studied is a patient initially diagnosed with VT by electrocardiogram and the model mainly uses cardiac ultrasound and laboratory data for further etiological diagnosis of VT. Another reason for applying cardiac ultrasound is that ultrasound is a simple, inexpensive, non-invasive, and reproducible imaging modality with a wide range of clinical applications [38]. Guidelines also advocate that structural heart disease should be ruled out by echocardiography and coronary artery disease in the diagnosis of the cause of ventricular arrhythmias and that further ancillary diagnostic tests should be performed at the discretion of the treating clinicians [39].

In this study, we compared explainable methods such as SHAP, LIME, and ICE and conducted a detailed analysis of their practicality in clinical settings [40]. SHAP is a game-theoretic method that provides both global and local explanations. It explains model behaviour by calculating the contribution of each feature to the prediction. In our study, global feature importance was calculated as the average of the absolute SHAP values for each feature across the entire independent test set, helping clinicians gain a deeper understanding of the overall model behaviour. In addition, we use the SHAP explanation force plot to explain how each feature and its value contribute to the VT etiology diagnosis. LIME is a local interpretable method that approximates the behaviour of complex models by generating perturbed data and training a simple interpretable model [41]. LIME emphasises local interpretability and is suitable for explaining individual predictions. In our study, LIME provided a detailed explanation for the etiologic diagnosis of specific VT patients, enabling clinicians to understand the specific impact of each feature on a particular prediction. The ICE method visualises the change in predictions for each patient instance as a feature varies. Unlike PDP, ICE plots focus on the individual conditional expectations of each instance rather than the overall average effect. This approach helps clinicians understand how the features of specific patients influence the model’s predictions. Currently, these interpretable methods have become popular tools for interpreting machine learning models [42]. Among them, LIME and SHAP are widely used frameworks for model interpretability. We expect that these interpretable methods will help clinicians gain a deeper understanding of the decision-making process of the models, thus improving the acceptability and credibility of CDSS.

Despite the promising results of some interpretable methods demonstrated in this paper, in clinical practice, these methods can only serve as a supplement to data-driven models in enhancing the transparency of clinical decision-making [43]. They cannot replace the unique advantages of knowledge-driven models in terms of interpretability and evidence-based nature. Furthermore, due to the inherent limitations of the internal models of knowledge-driven systems, they cannot achieve the precision and personalisation offered by data-driven models [44]. Therefore, rule-based systems and machine learning models are complementary to each other, and a system that integrates both approaches may facilitate the adoption of artificial intelligence in healthcare applications [45].

An ideal Clinical Decision Support System (CDSS) should provide decision explainability to clinicians [43,46]. In the healthcare sector, predictive solutions require an effective method to enhance their interpretability [47,48]. Improving model interpretability not only allows for a better understanding of the model itself but also increases the utility of the model’s output, ultimately leading to improved patient outcomes [49,50]. With advancements in explainable machine learning tools, such as SHAP, XAI is rapidly maturing and becoming a powerful aid in clinical decision-making [51]. XAI enhances the intelligence provided to users by offering explanations, thereby increasing the transparency of results and conclusions. Although XAI has the potential to assist human decision-making through data-driven methods, there are challenges in explaining and interpreting the AI algorithms that transform system inputs into recommended outputs [52]. Currently, in medical practice, knowledge-based rule systems are more readily accepted by clinicians due to their clear logic. However, due to the inherent limitations of the internal models of knowledge-driven systems, they cannot achieve the precision and personalisation offered by data-driven models [44]. Therefore, rule-based systems and machine learning models are complementary to each other, and a system that integrates both approaches may facilitate the adoption of artificial intelligence in healthcare applications [45]. Each of the two driven CDSS has its own strengths and limitations, which drive the need for hybrid models that aim to combine the benefits of both. In 2017, the Defense Advanced Research Projects Agency (DARPA) proposed an initiative to integrate expert-knowledge-based and AI decision strategies. Several studies have also explored how to improve its accuracy while maintaining interpretability and validate it in the field of cardiovascular risk assessment decision-making [53]. Our future work will also increase the exploration of novel approaches in the area of interpretable clinical decision-making, aiming to make CDSS more transparent and efficient by fusing the strict logical rules of traditional knowledge-driven models with the powerful self-learning capabilities of intelligent algorithmic models.

Furthermore, given the primacy of transparency, rationality, and comprehension of decision-making pathways in high-stakes disciplines like cardiology [54], this study focuses on an XAI framework for the etiological diagnosis of VT, as well as assessment scenarios. The interpretable evaluation framework of this study draws on the principles of evaluating XAI for clinical decision-making. The results of our analyses remain subjective but can serve as a feasible underpinning for the initial exploration of interpretability for decision-making. Future work will continue to explore in depth the development of generalised assessment protocols for evaluation from a variety of XAI techniques in a clinical setting, making them a fundamental research tool for the future design of AI-driven CDSS.

### Limitations and Further Work

Our current work included data from 1305 patients in the arrhythmia unit of a central hospital; however, the results may not be generalisable. The application of interpretable methods to clinical decision-making needed to be assessed in more rigorous controlled trials at that time. Additionally, the validity of XAI in other disease areas required further exploration.

Currently, the best interpretable methods and visualisation approaches should be evaluated by prospective studies. Future work will involve inviting clinicians to use different XAI tools for clinical decision-making in real clinical scenarios. This will allow for an assessment of the efficiency of interpretable methods and the overall satisfaction of clinicians with the various XAI tools that assist in clinical decision-making, as well as to measure the impact on decision-making time.

## 5. Conclusions

In conclusion, we constructed an interpretable machine learning model for the etiological diagnosis of VT and proposed an evaluation framework. The XGBoost model demonstrated superior performance in the etiological diagnosis of VT, and the SHAP local interpretable method and decision tree global interpretable method are more favoured by clinicians for decision-making. Our work highlights the critical role of XAI in clinical decision-making in the etiological diagnosis of VT. Future work will continue to explore clinical decision-making methods that ensure both high accuracy and strong interpretability.

## Figures and Tables

**Table 1:** Baseline characteristics.

	All Patients (N = 1305)	Ischemic Heart Diseases (N = 253)	Non-Ischemic Structural Heart Diseases (N = 681)	Idiopathic VT (N = 371)
Demography				
Age	42.19 (17.35)	59.73 (9.90)	42.60 (15.68)	29.53 (13.10)
Females	19.81%	18.06%	23.27%	9.12%
BMI	24.69 (4.04)	25.97 (3.17)	24.60 (4.10)	24.00 (4.26)
Medical history				
HT	23.26%	6.98%	19.36%	56.80%
AP	15.52%	0.71%	4.74%	62.50%
HLP	20.69%	6.47%	14.39%	58.50%
DM	9.79%	1.16%	6.72%	30.04%
Smoke	40.70%	29.57%	36.23%	67.36%
Alcohol	31.67%	27.76%	30.59%	40.32%
Echocardiography				
MVv	0.81 (0.21)	0.78 (0.23)	0.80 (2.21)	0.84 (0.18)
LAAPd	35.79 (7.02)	40.87 (6.36)	36.17 (6.92)	31.66 (4.82)
LVEF	56.84 (11.89)	49.90 (11.26)	54.97 (12.35)	64.95 (5.17)
LVPWd	9.02 (1.64)	9.13 (1.59)	9.24 (1.83)	8.55 (1.17)
AscAo	30.20 (4.66)	33.64 (4.02)	30.12 (4.45)	28.09 (4.06)
PGMV	2.79 (1.51)	2.65 (1.69)	2.75 (1.51)	2.95 (1.37)
TVv	1.20 (0.38)	0.54 (0.11)	0.56 (0.14)	0.61 (0.12)
ARv	1.21 (0.30)	1.21 (0.24)	1.22 (0.35)	1.19 (0.21)
AoSV	31.19 (4.06)	33.56 (3.60)	30.90 (4.10)	30.20 (3.70)
PAd	22.97 (3.05)	24.09 (2.89)	23.19 (3.10)	21.25 (2.70)
LVIDd	51.39 (8.48)	57.07 (7.58)	52.26 (8.89)	46.01 (4.13)
PGTV	1.35 (0.88)	1.21 (0.53)	1.31 (1.07)	1.52 (0.66)
IVSd	9.62 (2.61)	9.87 (1.92)	10.02 (3.23)	8.76 (1.24)
AoA	21.81 (2.32)	22.66 (2.40)	21.70 (2.28)	21.47 (2.20)
Laboratory				
FT3	3.11 (0.76)	2.90 (0.59)	3.05 (0.66)	3.34 (0.97)
NT-proBNP	522.48 (1187.58)	780.26 (1676.63)	677.76 (1335.80)	166.90 (302.82)
LDL-C	2.51 (0.85)	2.04 (0.85)	2.59 (0.84)	2.69 (0.76)
K	4.00 (0.36)	4.12 (0.40)	4.05 (0.37)	4.06 (0.31)
HbA1C	5.85 (0.95)	6.53 (1.19)	5.83 (0.91)	5.44 (0.47)
Neut#	4.93 (1.94)	5.50 (2.02)	5.36 (1.90)	3.77 (1.41)
Glu	6 (1.62)	6.18 (1.92)	5.39 (1.38)	6.16 (2.32)
RBC	4.73 (0.64)	4.53 (0.64)	4.62 (0.64)	5.06 (0.51)
T4	8.22 (1.98)	8.41 (2.00)	8.31 (2.11)	7.94 (1.67)
BUN	5.57 (2.12)	6.38 (2.65)	5.50 (2.19)	5.16 (1.26)
Ca	2.17 (0.25)	2.06 (0.25)	2.07 (0.25)	2.36 (0.12)
ESR	6.00 (7.90)	8.42 (11.91)	5.75 (7.06)	4.40 (4.89)
Uric	341.71 (97.57)	329.88 (95.81)	335.34 (101.13)	361.51 (89.10)
T3	1.04 (0.26)	0.99 (0.14)	1.01 (0.23)	1.13 (0.32)
CK	90.10 (66.17)	90.37 (56.25)	86.74 (75.33)	96.13 (52.76)
PLT	210.39 (56.69)	193.62 (55.14)	201.21 (52.66)	238.73 (54.83)
apoA1	1.20 (0.38)	1.13 (0.34)	1.17 (0.37)	1.29 (0.25)
FT4	1.22 (0.36)	1.19 (0.39)	1.22 (0.42)	1.22 (0.22)
CHOL	4.17 (1.00)	3.68 (1.01)	4.26 (0.99)	4.5 (0.91)
TG	1.54 (1.13)	1.53 (0.95)	1.53 (0.94)	1.57 (1.52)
hsCRP	1.90 (2.78)	2.49 (3.54)	1.84 (2.68)	1.61 (2.28)
CRP	4.35 (10.39)	5.89 (13.72)	4.71 (11.87)	3.12 (3.39)
WBC	7.48 (2.12)	7.99 (2.23)	7.85 (2.12)	6.45 (1.66)
HDL-C	1.15 (0.34)	1.08 (0.29)	1.16 (0.38)	1.17 (0.31)
Lymph#	1.92 (0.67)	1.80 (0.70)	1.85 (0.66)	2.13 (0.64)
HGB	143.14 (17.36)	137.49 (15.53)	140.36 (17.41)	152.12 (14.96)
TSH	3.26 (5.69)	3.40 (5.18)	3.57 (6.96)	2.60 (2.43)

Notes: For categorical variables, percentages are presented, for scalar variables, median (interquartile range) is presented. HT: Hypertension; AP: Angina Pectoris; HLP: Hyperlipidemia; DM: Diabetes Mellitus; Smoke: Smoking History; Alcohol: Alcohol History; MVv: Mitral Valve Forward Flow Velocity; LAAPd: Left Atrial Anteroposterior Diameter; LVEF: Left Ventricular Ejection Fraction; LVPWd: Left Ventricular Posterior Wall Thickness in Diastole; AscAo: Ascending Aorta Diameter; PGMV: Pulmonary Gradient of Mitral Valve Forward Flow; TVv: Tricuspid Valve Forward Flow Velocity; ARv: Aortic Regurgitation Velocity; AoSV: Aortic Sinus Diameter; PAd: Pulmonary Artery Diameter; LVIDd: Left Ventricular Internal Diameter in Diastole; PGTV: Pulmonary Gradient of Tricuspid Valve Forward Flow; IVSd: Interventricular Septum Thickness in Diastole; AoA: Aortic Annulus Diameter; FT3: Free Triiodothyronine; NT-proBNP: N-terminal Pro-B-type Natriuretic Peptide; LDL-C: Low-Density Lipoprotein Cholesterol; K: Potassium; HbA1C: Glycated Hemoglobin; Neut#: Neutrophil Count; Glu: Glucose; RBC: Red Blood Cell Count; T4: Total Thyroxine; BUN: Blood Urea Nitrogen; Ca: Calcium; ESR: Erythrocyte Sedimentation Rate; Uric: Uric Acid; T3: Total Triiodothyronine; CK: Creatine Kinase; PLT: Platelet Count; apoA1: Apolipoprotein A1; FT4: Free Thyroxine; CHOL: Total Cholesterol; TG: Triglycerides; hsCRP: High-Sensitivity C-Reactive Protein; CRP: C-Reactive Protein; WBC: White Blood Cell Count; HDL-C: High-Density Lipoprotein Cholesterol; Lymph#: Lymphocyte Count; HGB: Hemoglobin; TSH: Thyroid-Stimulating Hormone.

**Table 2:** Performance of machine learning models for VT etiological diagnosis.

Model	Ischemic Heart Diseases			Non-Ischemic Structural Heart Diseases			Idiopathic VT		
	P	R	F1	P	R	F1	P	R	F1
Logistic Regression	0.735	0.800	0.766	0.562	0.360	0.439	0.764	0.824	0.824
Random Forest	0.884	0.884	0.884	0.746	0.707	0.726	0.934	**0.971**	**0.952**
XGBoost	**0.879**	**0.916**	**0.897**	0.809	**0.733**	**0.769**	**0.950**	0.931	0.941
LightGBM	0.879	0.912	0.895	**0.815**	0.707	0.757	0.933	0.951	0.942
SVM	0.840	0.879	0.859	0.823	0.680	0.745	0.848	0.873	0.860

Notes: Bold indicates the highest value for each indicator.

**Table 3:** Comparison of overall model performance.

Model	P	R	F1
Logistic Regression	0.709	0.722	0.710
Random Forest	0.871	0.872	0.871
XGBoost	**0.884**	**0.885**	**0.884**
LightGBM	0.881	0.883	0.881
SVM	0.839	0.839	0.837

Notes: Bold indicates the highest value for each indicator.

**Table 4:** Evaluation of interpretable methods for clinical decision-making.

Methods	Visualisation			Clinical Usability	Clinical Applicability	
	Form	Information Volume	Difficulty Level		Global	Local
Decision rules	Text	Low	Low	Medium	√	
Decision tree	Graphs, tree diagrams	Medium	Low	High	√	
PDP, ICE	Graphs, line diagrams	High	Medium	Low	√	
LIME	Graphs, bar charts	Medium	Low	Medium		√
SHAP	Graphs, dot/bar charts	High	High	High	√	√

**Table 5:** Results of feature ablation based on XGBoost model.

Feature	P	R	F1
AP	0.830	0.829	0.830
age	0.884	0.875	0.879
HLP	0.876	0.878	0.876
LVEF	0.883	0.865	0.873
NT-proBNP	0.882	0.866	0.873
LVIDd	0.894	0.881	0.886
LDL-C	0.889	0.875	0.882
AoSV	0.875	0.858	0.866
hsCRP	0.880	0.864	0.871
HT	0.871	0.860	0.865
HbA1C	0.879	0.866	0.872
LAAPd	0.870	0.866	0.868
Glu	0.873	0.852	0.862
CHOL	0.882	0.865	0.873
ARv	0.863	0.857	0.860
CK	0.864	0.860	0.862
HGB	0.879	0.868	0.873
PLT	0.878	0.863	0.870
ESR	0.874	0.868	0.871
TSH	0.888	0.867	0.877

## Data Availability

The data presented in this study are available on request from the corresponding author due to (specify the reason for the restriction).
